# Outlining recent updates on influenza therapeutics and vaccines: A comprehensive review

**DOI:** 10.1016/j.jvacx.2024.100452

**Published:** 2024-01-30

**Authors:** Shiza Malik, Muhammad Asghar, Yasir Waheed

**Affiliations:** a Bridging Health Foundation, Rawalpindi, Punjab 46000, Pakistan; b Department of Biology, Lund University, Sweden; c Department of Healthcare Biotechnology, Atta-Ur-Rahman School of Applied Biosciences (ASAB), National University of Sciences and Technology (NUST), H-12, Islamabad, Pakistan; d Office of Research, Innovation, and Commercialization (ORIC), Shaheed Zulfiqar Ali Bhutto Medical University (SZABMU), Islamabad 44000, Pakistan; e Gilbert and Rose-Marie Chagoury School of Medicine, Lebanese American University, Byblos 1401, Lebanon

**Keywords:** Influenza virus, Influenza virus infection, Therapeutics, Antiviral agents, Vaccines, Treatment, Novel therapeutic, Clinical management

## Abstract

Influenza virus has presented a considerable healthcare challenge during the past years, particularly in vulnerable groups with compromised immune systems. Therapeutics and vaccination have always been in research annals since the spread of influenza. Efforts have been going on to develop an antiviral therapeutic approach that could assist in better disease management and reduce the overall disease complexity, resistance development, and fatality rates. On the other hand, vaccination presents a chance for effective, long-term, cost-benefit, and preventive response against the morbidity and mortality associated with the influenza. However, the issues of resistance development, strain mutation, antigenic variability, and inability to cure wide-spectrum and large-scale strains of the virus by available vaccines remain there. The article gathers the updated data for the therapeutics and available influenza vaccines, their mechanism of action, shortcomings, and trials under clinical experimentation. A methodological approach has been adopted to identify the prospective therapeutics and available vaccines approved and within the clinical trials against the influenza virus. Review contains influenza therapeutics, including traditional and novel antiviral drugs and inhibitor therapies against influenza virus as well as research trials based on newer drug combinations and latest technologies such as nanotechnology and organic and plant-based natural products. Most recent development of influenza vaccine has been discussed including some updates on traditional vaccination protocols and discussion on next-generation and upgraded novel technologies. This review will help the readers to understand the righteous approach for dealing with influenza virus infection and for deducing futuristic approaches for novel therapeutic and vaccine trials against Influenza.

## Introduction

Influenza viruses cause significant global health burden, and are highly contagious and easily spreading virus, which can lead to epidemic and even pandemic [1]. Influenza viruses causes a mild disease, characterized by fever, nasal congestion, cough, and muscle aches, and does not require specific therapeutics for cure[2]. However, severe disease mainly affects infants, pregnant women, and the elderly or immunocompromised individuals [3], [4], [5]. Annually one billion influenza cases occurs, resulting in approximately 3–5 million sever cases and around half a million death [4], [5], [6], [7], [8]. Influenza virus mainly transmitted by inhalation of viral particle suspended in aerosol or droplets and target the upper respiratory epithelial cells [6], [7], [8].

Biologically influenza virus is an RNA virus with eight segmented negative sense strains of RNA incorporated within a lipid envelope [9], belongs to family *Orthomyxoviridae* and contain at least three subtypes A, B, and C virus (IAV, IBV and ICV, respectively) [10]. However, influenza virus rapidly mutates and has ability develop resistance against the antiviral compounds via different processes like antigenic shift (genome segment reassortment), and antigenic drift (genetic mutation) resulting in a new clads and subclades of influenza virus [11], [12]. One such mutant type; caused by antigenic shift (genome segment reassortment), was notified back in 2009, namely, the H1N1 subtype of influenza that emerged from swine and caused an epidemic [13]. Such genetic variations cause the frightening possibility of H5N1 avian flu causing viruses to gain the function of transmissibility between humans. Similarly, the rise of other avian influenza virus H5N1 (bird flu), H1N1 (swine flu) and H3N2 have caused significant health burden [14], [15], [16], [17], [18]. Thus making it pertinent for the scientific community to control their further spread and confine their proliferation among humans through the development of appropriate therapeutics against different strains [18], [19], [20], [21].

Several therapeutic trials have been conducted in the past targeting influenza virus replication cycles, enhancing immune system responses, resisting the mutational abilities of viruses, and overcoming the resistance that develops against therapeutics in influenza viruses [22]. A few candidate therapeutics have been approved by the Food and Drug Administration (FDA) such as amantadine, rimantadine, zanamivir, oseltamivir [21], [23], while others are still in trails. These approved drugs mostly inhibits virus replication process within the host cell [24], [25], [26], [27], [28] and reduce disease symptoms and severity. However, the influenza viruses have shown to slowly develop resistance against these drugs, which limits the time window and efficacy profile of approved drugs [21], [21], [29].

Vaccination against viral diseases is a cost-effective, efficacious, and rapid counter-response to control epidemic and pandemic [30]. However, the strain-specific immunity and continuous viral genetic mutation remain a hurdle in universal vaccine design, especially in the case of the influenza virus [31], [32]. The major structural features inquired in influenzas that make the vaccination efforts practical include the surface proteins hemagglutinin (HA), which induces fusion of the virus to sialic acid-containing receptors in host cells after the virion has attached to the cell surface and been endocytosed. The fusion process is how the HA fuses the virus envelope and the endosomal membrane to break it apart and release the virion's genetic segments into the cell's cytosol [33], [34]. While the 2nd category of surface proteins neuraminidase (NA) acts as a receptor destroyer enzyme and plays roles in viral release and cellular transmission. Influenza A virus is further divided into subtypes based on which HA (out of 18) and NA (out of 11) they display on surface of their viral membrane (e.g., H1N1, H3N2, H5N1).

[10], [33], [34]. The currently licensed vaccines against influenza work either on the principle of inactivated or live attenuated vaccination protocols or those devised on principles of subunit vaccines which majorly incorporate the influenza viral particles such as surface proteins HA, and NA fragments [33], [35], [35]. Moreover, the vaccination design may involve two or three viral species leading to formulations of divalent, trivalent, and quadrivalent vaccine formulations incorporating multiple strain fragments [36], [37], [38]. It should be noted that the WHO has not detected any Yamagata lineage of influenza B since 3/2020 so it currently is recommending that this strain not be included in seasonal vaccine formulations [38]. In this review, we have tried to briefly describe recent updates on influenza therapeutics and some of the important vaccination protocols and associated ongoing research trials worldwide.

## Methodology

### Search Criteria

A methodological research strategy has been adopted for this literature review to include data from diverse, recent, and most cited sources of research studies. Data was collected via a methodological literature search through various online sources including Google Scholar, PubMed, NIH (National Library of Medicine), Web of Science, European database, Springer, and Embase databases. Statistical and epidemiological results have been gathered from the official websites of the WHO, CDC, and FDA. The study inculcates original research articles, sections from books, letters to the editors, short and lengthy reviews, and some case studies published recently. For data on therapeutics against Influenza viruses, major search terms were “influenza virus”, “influenza virus disease”, “therapeutics against influenza”, “antiviral agents,” “vaccines against influenza”, “therapies against influenza”, “novel therapeutic approaches” while for vaccines against Influenza viruses, major search terms were “influenza virus”, “influenza virus biology”, “vaccines against influenza”, “novel vaccination trials” and “therapies against influenza”.

### Inclusion and exclusion strategy

The inclusion and exclusion criteria simply revolved around gathering data from original research articles, sections from books, letters to the editors, short and lengthy reviews, and some case studies published recently. The available data were immense, but we tried limiting the data to around 150 studies, from which the interlinked data have been gathered. Thus, after a thorough analysis of the dates, abstracts, titles, and journals of research publications, they were included in this review. Moreover, this review has mostly reviewed studies conducted after 2010, to elaborate on the latest therapeutic interventions and vaccines developed in the past decade. It should be noted that studies of English origin have been made part of the review.

## Results

### Chronic influenza infection and need for Therapies?

There exist four categories of influenza viruses: A, B, C, and D. worldwide, nearly every winter, influenza A and B viruses lead to seasonal outbreaks of illness in humans, commonly referred to as the flu season [3], [4], [5], [6], [7]. Influenza A viruses stand as the exclusive culprits behind flu pandemics, marked by worldwide outbreaks of flu-related illnesses. A pandemic scenario unfolds when a novel and distinct influenza A virus emerges, capable of infecting humans, efficiently spreading among them, and encountering limited immunity [9], [12]. On the other hand, Influenza C virus infections generally result in mild sickness and are not deemed responsible for widespread human outbreaks. In contrast, Influenza D viruses primarily impact cattle, occasionally spilling over to affect other animals, yet lacking the propensity to induce illness in humans [12], [13], [14], [15], [16], [17]. Influenza mostly causes mild infection of the upper respiratory tract [39]. However, in elderly or immune-compromised individuals can develop a severe infection and chronic cases in which the virus survives, especially in, where immunopathogenesis by influenza results in direct cellular damage in the airway epithelial layer coupled with tissue damage to the associated organs [40]. During this period the influenza load peaks in the upper tract and peaks at the 3rd day causing fever and clinical symptoms. After 3rd-day the immune system mostly deals with the viral replication rates and viral loads and thus drops down [41], [42].

**Fig. 1:**
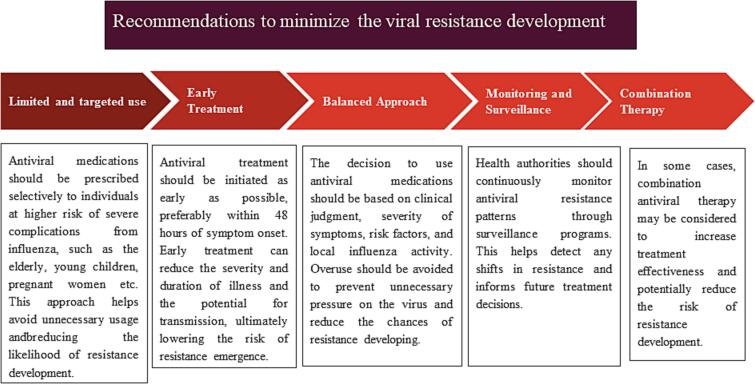
Recommendations to minimize the viral resistance development.

**Fig. 2:**
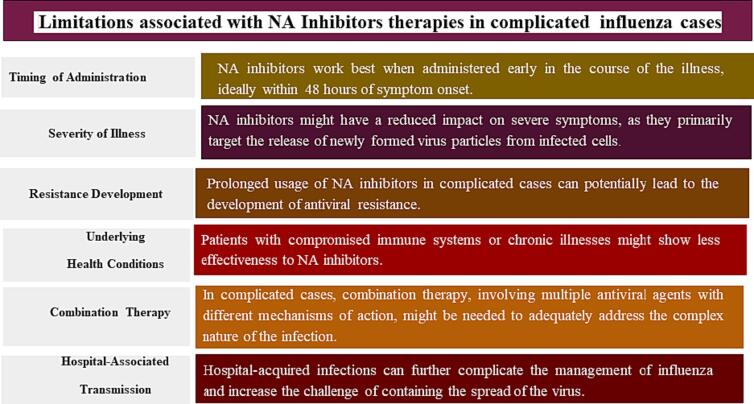
Limitations associated with NA Inhibitors therapies in complicated influenza cases.

This viral unloading, immune regulation, and controlled disease outcomes present an opportunity for scientists to design therapeutics to regulate the immune mechanism of action, especially to prevent cases of rapid disease progression and complications associated with chronic influenza infection [13], [24], [43], [44], [45]. In most cases, treatment with different therapeutics is initiated before the virus takes a peak account within the body (24–36 h) [20]. The purpose is to strengthen the immune system and fight against influenza infection. Various disease management protocols and disease mitigation measures have indicated that a proactive response and rapid diagnosis and start of treatment are paramount to the therapeutic advantage against infection [11], [46]. Thus, it will not be wrong to say that treatment-initiated time, body conditions, age, and immune system characteristics affect the treatment outcomes against Influenza infection [9], [34], [47], [48]. Moreover, several recommendations have been suggested by the World Health Organization (WHO)to devise treatments for healthy people for the concerns of minimizing the viral resistance development [49], [50], [51]. These guidelines suggest the use of antiviral medications, such as oseltamivir and zanamivir, for the treatment of confirmed or suspected cases of influenza. However, WHO emphasizes responsible usage to prevent the emergence of drug-resistant strains. These recommendations have been compiled in a figure format (Fig. 1) in the following section. Some of the proposed trials and approved candidates of therapeutics against influenza are covered in the following sections and are presented in Table 1.Fig. 2..

**Table 1:** Summarizing therapeutics against influenzas virus.

**Sr. No.**	Therapeutic Strategies	**Names Of Respective Antiviral Agents**	**References**
1	NA Inhibitors	● AdamantanesZanamivir (Relenza)Oseltamivir (Tamiflu) Peramivir	[52], [53], [54], [60], [69], [53], [141]
2	Long-Acting NA Inhibitor	● laninamivir (R-125489Laninamivir octanoate (CS-8958) prodrug	[55], [56], [58], [59]
3	M2 Ion Channel Inhibitors	● Amantadine (Symmetrel)Rimantadine (Flumadine)	[79], [80]
4	Polymerase Inhibitors	● Favipiravir (T-705)Pimodivir (VX-787) Combination of oseltamivir and T-705 Ribavirin	[60], [61], [62], [64], [65], [142]
5	HA Inhibitor	● Cyanovirin-N (CV-N),	[55], [60], [69], [43]
6	Sialic Acid Receptor Inhibitor	● DAS181 (Fludase®)	[51], [64], [67], [70], [77], [78], [143]
7	PA Endonuclease Inhibitors	● Baloxavir Marboxil(PA I38T/M/F) model experiment EIDD-2801	[63], [64], [66], [77], [78], [143]
8	Neutralizing Antibodies (nAbs)	● MHAA4548A, MEDI8852, VIS410	[74], [76], [144], [145], [146], [147], [148]
9	Next-Generation Influenza Virus Inhibitor	● Prodrug EIDD-2801, a 5′-isopropylester	[14], [50], [73], [77], [81], [50], [149]
10	Combination Therapy	● Adamantanes (amantadine and rimantadine) + ribavirin. Rimantadine + NA inhibitor (zanamivir, oseltamivir carboxylate, or peramivir)Triple combination antiviral drug (TCAD) regimen (oseltamivir carboxylate, amantadine, and ribavirin) Peramivir + Ribavirin Rimantadine + Oseltamivir Oseltamivir + Ribavirin Oseltamivir + Amantadine Or Rimantadine Amantadine + Oseltamivir	[18], [20], [21], [22], [24], [33], [45], [46], [82], [83], [84], [12], [144], [150], [151], [26], [152], [153]
11	Other drug candidates	● Systemic steroids and corticosteroids Drugs Statins Fibrates Thiozolidinediones Cyclooxygenase Pathway InhibitorsCorticosteroids (Anti-Inflammatory) Antioxidant- N-Acetyl-L-CysteineRosuvastatin (Cholesterol Biosynthesis)	[3], [24], [40], [71], [72], [73], [149], [154]
12	FDA approved drugs	● Baloxavir marboxil Oseltamivir Peramivir Zanamivir Amantadine Rimantadine Favipiravir	[9], [26], [27], [46], [47], [48], [89], [144], [150], [1], [155]

## Modern therapeutic strategies against chronic and seasonal influenzas

### Parenteral neuraminidase NA inhibitors and long-acting NA inhibitors

NA inhibitors are the most widely used anti-influenza agents that inhibit the enzymatic activity of neuraminidases of Influenza A and Influenza B viruses (IAV) and (IBV) [52], preventing the cleavage in sialic acid residues on viral and host cell surfaces, blocking the virions binding on the host cell surface, resulting in virion replication cycle arrest [53], [54]. Zanamivir (Relenza) and Oseltamivir (Tamiflu) are two of the antiviral medications used to treat and provide prophylaxis against influenza A and B. These drugs work by binding to neuraminidase, preventing the virus from escaping its host cell and infecting others.

NAI has proven efficacy against influenza infections, but also has shown limitation against complicated and hospitalized cases [55]. Addressing these limitations requires a multifaceted approach, including early diagnosis, judicious use of antiviral medications, potential combination therapy, and infection control measures to prevent further transmission. Some of the limitations have been described in figure-2. Resistance to NAI by numerous influenza strains is well documented and is an increasing problem The problem of rapid resistance development against NA proteins (H274Y, I223R, E119V, R292K, and N294S, Q136K) has been identified in NA inhibitors therapies that work against specific influenza subtypes such as H1N1, H5N1, and H3N2 subtypes [55], [56], [57].

Some studies suggest that approximately 90 % of strains of influenzas viruses developed resistance to these drugs during Pandemic times [51], [55], [57], [58], [59]. To overcome this challenge a new class of NA inhibitors has been introduced during endemic 2009, known as Long-Acting NA Inhibitors, which prolong the retention of drugs in the lungs and requires lower drug dosages and greater efficacy compared to the previous class of NA inhibitor drugs [55]. The designed drugs showed impact against common influenza viruses, viral subtypes N1-N9, and H1N1, H5N1 [56], [58].

### RNA polymerase and polymerase complexes inhibitors

These drugs work by inhibiting viral replication machinery during the early stages of the replication cycle by inhibition of RNA polymerase. The polymerases are incorporated into the newly synthesized viral RNA, resulting in transitioning mutation and collapse of the viral replication cycle [60]. These antiviral drugs have shown efficacy against various types of influenza viruses, including influenza species A (subtypes: H1N1, H2N2, and H3N2), B, and C and oseltamivir-resistant viruses [60], [61]. A dose-dependent application of these drugs has been checked to reduce virus titers to event fatality and to inhibit lung consolidation, thus, presenting a chance to overcome the resistant nature of the influenza virus and reduce modalities associated with hospitalized cases [62], [63]. Notably, a similar mechanism of action has been proposed for other viruses such as arenavirus, bunyavirus, flavivirus, norovirus, picornavirus, alphavirus, paramyxovirus, and rhabdovirus families [64], [65]. Similar to the polymerase inhibitor mechanism, some antiviral agents are being designed against RNA-dependent RNA polymerase (RdRP) complexes of infectious viruses. These are small-scale molecular therapeutics that are radially tested in clinical trials for their associated efficacy. The replication strategy associated with RdRP organization and polymerase complex is majorly conserved among subtypes of IAVs and IBVs, which increases the likelihood of the development of a broad-spectrum anti-therapeutic approach against different influenza species [63], [66].

### HA Inhibitors

HA inhibitors function by binding to the glycoproteins of enveloped viruses, undergoing structural changes, and inhibiting viral entry into the host cell [53], [67]. Moreover, the mutational problem associated with glycosylation sites of HA further reduces the antiviral activities. Since the specific strains of influenza viruses can change from season to season due to antigenic drift (minor genetic changes), and occasionally antigenic shift (major genetic changes), therefore, more work is needed to prove their anti-viral efficacy against a diverse range of influenza species and to establish modified versions of HA inhibitors against mutated regions [2], [12], [46], [68], [69].

### Sialic acid receptor inhibitors

This drug inhibits the host cell components necessary for viral replication and reduce the drug resistance variants among influenza viruses in the host [67]. Receptors containing sialic acid (SA) are present in susceptible host cells essential for the binding of HA viral proteins with host receptor cells [51]. These antiviral agents avert infection from influenza A (H1N1, H5N1), B virus subtypes, and NA inhibitor-resistant influenzas species, and parainfluenza viruses[51], [67], [70]. Although the cytotoxicity profile of Sialic Acid Receptor Inhibitors are well established, its resistive profile needs to be further established in human subjects [51].

### Neutralizing antibodies (nAbs)

Antibodies-based therapy have shown high tolerability and enhanced pharmacokinetic against influenza infections [71], just like neutralizing antibodies treatment against different viral infections such as Ebola, RSV, HIV, Dengue, and others. In the case of an influenza virus, the nAbs function against the highly immunogenic receptor binding sites (RBCs) across different subtypes of the influenza virus of the virus [3], [72], [73]. Neutralizing antibodies accelerate the resolution of disease severity and reduce viral load in the tested models. Moreover, an essential feature is their long shelf life and reduced dosage needs, with no reported resistance reported yet [74], [75]. Some novel research is coming forward that includes the use of multidomain antibodies against different types of influenza viruses [71], [74], but need further assessment against cross-resistance and cost-benefit analyses, as these therapies are still in research phase 2 [74], [76].

### PA endonuclease inhibitors

These inhibitor drugs are a new class of anti-influenza agents that blocking the PA endonuclease activity, necessary for viral infectious cycle regulation [63]. Their efficacy is similar to standard therapy approaches with NAIs but presents better dose efficacy compared to NAIs [63], [66], [77], [78]. Moreover, the drugs in this category, specifically baloxavir marboxil has shown antiviral activity against various subtypes of influenza virus, including A, B, C, and G generas, making them a wide spectrum of therapeutics against influenza infection [[66]. There are some minor side effects associated with the drugs, such as headache, increased white blood cell count [13], [66]. Nevertheless, resistance has also been observed in some patients against baloxavir marboxil, with a rebound viral load and the prolonged manifestation of clinical symptoms associated with patients’ profiles [13], [66].

### M2 ion channel inhibitors

These antiviral agents function by inhibiting the replication cycle at two different steps of viral replication. These drugs block the ion channel activity of M2 proteins of IAVs, which is necessary for virus entry and uncoating within the host cell as well as inhibit activity at the later stages of replication, preventing the release of virion replicons from the infected host cells [[79], [80]. Moreover, this replication occurs in a strain-specific manner.

### Next-generation influenza virus inhibitors

These antiviral agents use a novel approach that involve efficient anabolism of the bioactive tri-phosphate component, followed by incorporation as cytisine into the viral RNA with the help of polymerase action [50]. This is followed by high rates of transition mutation in viral RNA polymerase and other transcription products resulting errors in replication cycle and lower viral loads in tissue cultures [[81], [50]. These antiviral strategies have been checked against other infectious viruses belonging to the alphavirus, coronavirus, flavivirus, and togavirus families, making them a possible candidate for broad-spectrum antiviral agents. However, to establish their safety profiles these antiviral candidates further clinical experiments on are needed [14], [73], [77], [50].

### Other potential drug agents in clinical trials

With the advancement in our understanding about influenza virus life cycle, replication machinery, mechanism of action toward disease development and the respective immune response from host cells new drug candidates come to the surface, such as small interfering RNAs (siRNAs) and micro RNAs (miRs) that could be designed to target viral RNA without affecting host RNA [11], [82]. These RNAs based technology has already shown its targeted efficiency in various diseases in terms of high specificity, efficiency, low toxicity, and ease of formulation [83], [84]. A diverse range of in silico experiments are in research annals for RNA-based inhibitor therapies against influenza virus infection, the need is to dictate these proposed candidates into clinical trials [85], [86].

Similarly, immune therapies are introduced in the market against several infectious viral diseases such as neutralizing antibodies *(nAbs)* against influenza viruses (as discussed above). These antibody-based therapies may include intravenous immune globulin (IVIG) (simple terms antibodies), hyperactive immune sera from vaccinated individuals, and mixtures of monoclonal antibodies for therapies [71], [74]. These immunomodulatory therapies broadly work to neutralize viral infection by reducing the inflammatory response and activating the immune systems against viruses’ entrance [74], [75]. Additionally, different chemical, hormones, and enzymatic preparations have also been studied in different clinical experiments, some of which have been made part of Table 1, but further clinical experiments on are needed.

## Current influenza vaccines

The ever-evolving nature of the Influenza virus makes it pertinent to design a permanent therapeutic solution [11], [22], [53], [82], [12]. The mutations are exhibited in the various virus proteins such as surface glycoproteins HA, NA, or nucleoproteins [71], [87], [88], [89], resulting in generation of new viral strains. Antigenic shift occurs when there is a reassortment of genetic segments between different virus strains. Typically, it is a transfer from animal (i.e. swine) into human strains. Pandemic strains emerge as a consequence of antigenic shift because it presents a new virus to a naive immune system.

Additionally, the point mutations result in major antigenic shifts and heterogeneity in virus strains and thus make it difficult to formulate a one-time solution against it [9], [10], [33], [89]. The various pandemics and epidemics of the past such as those caused by (H1N1-First human case in 1918, 2009; H2N2-1957; 1968-H3N2; H5N1: 1997; H7N9:2013) subtypes, presented a public healthcare threat worldwide [28], [34], [35], [90]. While Limited human cases reported, with no specific pandemic identified for H7N2, H7N3, H7N7 and H9N2. Several vaccines have been developed against influenzas virus and some of the latest vaccination approaches with established efficacious profiles are discussed below (see Table 2).

**Table 2:** Vaccination strategies against influenza virus.

**Sr. No.**	**Vaccines Category**	**Vaccine Candidates Under Clinical Trials**	**Reference**
1	HA protein-based vaccine	● HA Rosettes, HA nanoparticles, VLP + Matrix-MTM adjuvant HA stem or head-stem chimeraFlu Blok (recombinant) Monoglycosylated HA- a universal flu vaccine candidate, with conserved domain	[32], [35], [91]
2	Epitope-peptides based vaccine (NA or HA based vaccines)	● HA, NP, M1 peptides FLU-V MVA-vectored NP + M1 vaccine	[51], [52], [53], [54], [55], [56], [97], [98], [53], [156]
3	Live attenuated virus vaccine	● CodaVaxFluMist (AstraZeneca) M2SRFluenz Tetra (licensed)M2-deficient single replication virus (targeting B cells) Approved vaccines A/Len/134/17/57, A/Len/134/47/57, B/USSR/60/69, A/Ann Arbor/6/60, B/Ann Arbor/1/66	[35], [35], [109], [110], [111], [157], [158]
4	Nucleic acid-based vaccine	● Vaccines candidates belonging to DNA and Mrna targeting HA Nabs DNA based- mucosal influenza vaccines – FluMist	[17], [109], [122], [123], [124], [159], [160]
5	Virus like particles-based vaccines (recombinant technology)	● M2e5x VLP vaccinesM2 VLP-supplemented inactivated influenza virus vaccine (A/PR/8/34, H1N1)H1N1 split vaccine (A/California/07/2009) + M2e5x VLPsrecombinant vaccine candidate-M2e-HBc fusion protein (ACAM-FLU-A)M2e-flagellin fusion vaccine (STF2.4xM2e)Ferritin-based NPs–HA (targeting HA Nabs)VLP–HA; NA; M1; M2 (targeting HA NAbs; T cells)Peptide–HA, NP, M1 (targeting T cells; B cells)Peptide–NP; M1; M2 (targeting T cells)	[57], [100], [113], [115], [118], [161]
6	Vector based vaccines	● Alphavirus- (targeting HA Nabs)Adenovirus- (targeting HA Nabs) Chimpanzee adenovirus–NP + M2 (targeting T cells) Modified vaccinia virus Ankara–HA NP + M1 (targeting HA NAbs; T cells)	[131], [132], [148], [162]
7	M2-based protein vaccine	● M2e + HBVc VLPs Recombinant M2e-GCN4 conjugate vaccine	[79], [80], [114], [162]
8	Inactivated split virus vaccines (Targeting HAI and licensed)	● Afluria Fluarix FluLavel Fluzone, Fluzone HD Fluad Influvac, Imuvac Fluarix, Alpharix, Influsplit 3Fluart Afluria, Enzira Vaxigrip, Vaxigrip Tetra	[35], [35], [108], [114], [148], [163]
9	Inactivated Subunit vaccines (Targeting HAI and licensed)	● Fluvirin Flucelvax Agrippal Fluad	[35], [35], [114], [148], [163]
10	T cell-based vaccines	● (MVA-NP + M1)	[40], [119], [120], [149]
11	Trivalent and Quadrivalent vaccines	● MedImmune's nasal spray vaccine, FluMist quadrivalent injectable quadrivalent influenza vaccine.	[36], [37], [38], [112], [164]
12	Adjuvants for influenza vaccination	● Alum, MF59, AS03 and AF03, saponins,Toll-like receptor (TLR) agonists such as TLR4, TLR5, TLR7/8 and TLR9, Polysaccharides and glycolipids, lipopeptides, glycolipids, Nucleotides, small-molecule inhibitors Bacterial-derived components, such as flagellin **Natural compounds drved adjuvants** QS21, is a saponin-based adjuvant.Extract from *Panax ginseng*. Alpha-galactosyl ceramide (alpha-GalCer), an extract from marine sponge. **Cytokine adjuvants- inflammatory mediator** IL-6, GM-CSF **Synthetic nanocarriers and Cationic lipids (liposomes)** Vaxfectin -cationic lipid-based systemPoly(lactic-co-glycolic) acid, Chitosan, Polyethyleneimine.	[105], [131], [132], [133], [134], [160], [165], [166]

### Ha-based vaccines against influenza

HA is the type I transmembrane glycoprotein (surface glycoprotein) that presents a major fraction of enveloped viral proteins and help viral attachment and cellular entry [91]. In case of influenza infection or vaccine regimens it acts as antigens and help mediate the immune responses [92]. Neutralizing antibodies are produced that avert the viral entry and receptor-mediated endocytosis of the Influenza virus. The principle is in use for a long for traditional vaccine designs and differential characteristics are assigned to vaccine formulation by varying the antigenic HA components within vaccines [93], [94], [95]. Moreover, with the enhanced structural understanding of HA and other enveloped proteins through bioinformatics, biophysics, and biochemistry tools, better vaccine formulations are on board for the future [71], [96]. However, there remains the hurdle of virus strain-specific mutational alterations that render the HA-based vaccine ineffective against a broad range of viruses [91]. Several clinical trials based upon HA-based vaccination approaches have been conducted in recent years, some of which have been made part of table 2 [20], [21].

### Neuraminidase NA-Based vaccines

Neuraminidase, another influenza-enveloped protein that has remained in vaccination practices and have shown better immunoregulatory activity as compared to standard HA-vaccine [97]. NA is a type II transmembrane glycoprotein with further 11 subtypes divided into three genetic groups [52]. Like HA antigens, antibodies against NA have also been identified but their exact role in immune regulation remains elusive [52], [53]. Therapeutics and antiviral agents such as oseltamivir, zanamivir, and lopinavir have been designed to inhibit NA and the same principle is under investigation for vaccine designing [53], [54], [71], [88], [98]. Additionally, the research show that NA and HA mutation, and genetic diversity are irrespective of each other, and the confluence results in a lesser severity of viral mutation and better chances for therapeutic management against influenza [98], [53], [53]. Thus, several NA-based vaccine trials are under consideration (Table 2).

### Matrix protein 2 ectodomain-based vaccines

Matrix protein 2 ectodomains (M2e) is an important proteinaceous entity, which is a pH-dependent and proton-selective ion channel protein [99]. The proteins function to help the virus disassemble, and release of virus core and subsequent viral budding and release and thus are conserved in influenza viruses [99], [100]. However, it has shown lesser immunogenicity when exposed to a cellular environment. However, experiments based on additional adjuvants and combination therapies show that these particles could be utilized for broad-range immunity generation in animal models [101]. Although the quantification of M2e base vaccination regiments will remain a limitation when a universal vaccine candidate will be designed since it could only passively create monoclonal antibodies and decrease overall viral load in influenza incidences [102]. Some antiviral therapies such as amantadine and rimantadine against influenza infection are also based on inhibiting activity against M2 protein [103], [104]. Formulation of a broad spectrum and largely immunoregulatory vaccine by conjugating the M2e-based vaccine with HA-based vaccination, is going on.

### Nucleoprotein-based vaccines

NP is an internal influenza virus protein that serves as a target for generating T cell-based immune responses and thus is under various ongoing trials [105]. T cell-based responses cause stimulation of intraepithelial tissue-resident memory immune responses [103], [106], [107]. It holds the ability to generate durability, potency, and a wide breadth of immunity against influenza infection. Moreover, NP-based therapies have also exhibited the characteristic of heterosubtypic immunity regulation against influenza subtypes. T-cell-based immunity regulates direct cytotoxicity induced by CD-8 cell-mediated responses and generates B-cell responses in the form of an immune system interconnected response when exposed to influenza particles [101], [105]. Persons that undergo NP-based vaccination have shown lower symptoms of influenza as compared to unvaccinated. The need is to further carry out experiments in this domain to establish a universal vaccine.

### Conventional inactivated influenza vaccines

In this approach, chemically or radiationally inactivated virus particles are used which are unable to further divide when introduced into host cells, but cause antibodies-based immune responses [35], [108]. The mechanism was used for the first vaccine trials that involved a whole inactivated virus particle for both influenza A and B subtypes [35]. One of the currently used conventional vaccine candidates is a trivalent vaccine that contains B-type influenza variants and two of influenzas A variants (H1N1 and H3N2) [37]. They work on the same principle as the HA and NA-based vaccine protocol. Due to their highly efficacious nature, they are in practice since 1970. A limiting feature of these vaccines is their variable efficacy, short immune-protective regulation, and low capacity to induce cellular immunity [35], [108]. Thus, efforts should be diverted to conjugate these vaccines with other vaccine formulations and adjuvants for better treatment response.

### Live attenuated virus (LAIV) vaccines

**Fig. 3:**
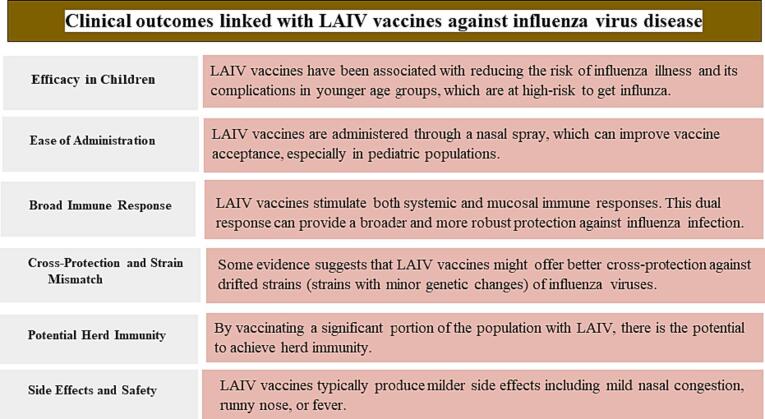
Clinical Outcomes Linked with LAIV Vaccines Against Influenza Virus Disease.

To deal with the variable efficacy of inactivated vaccines, another traditional approach of using live virus particles has been in practice. These vaccines are designed with the feature of internal proteins from a master donor virus and surface glycoproteins from wild-type influenza [109], [110]. They have shown better vaccination outcomes, especially in children as compared to inactive vaccine candidates, thus could be utilized against all children and adults below age of 50 years, and those with respiratory problems. Moreover, they induce humoral and cellular antibody-based immunity and Potential Herd Immunity in case of vaccination against a significant population of patients [35], [111]. Some clinical outcomes linked with LAIV vaccines against influenza virus disease are compiled in the Fig. 3 below.

### Trivalent and quadrivalent influenza vaccines

As elaborated earlier vaccine designs such as trivalent and quadrivalent are designed to curtail the wide spectrum of influenza infection. Quadrivalent vaccine design incorporates four strains, two from influenza A subtype (H1N1, H3N2) and two from type B strains (Victoria, Yamagata) [37]. These vaccination designs are useful since the virus keeps on mutating and generating different strains. Thus, multiple trivalent and quadrivalent vaccine trials are under clinical experimental design phase. In case of trivalent inactivated vaccine candidates, three vaccine subtypes conjugates: spilled, subunit, and whole virus vaccines are used [37], [38]. They are often used together to induce more specific and highly immunogenic responses. In the case of split vaccines, the virus is rendered inactive, in the subunit, the HA or NA antigens are used [36], [37], [112]. Moreover, these vaccines involve conjugation with adjuvants.

Trivalent influenza vaccines typically contain three strains of influenza virus: two subtypes of influenza A and one influenza B strain. The composition of these strains is determined based on predictions about the most likely circulating flu viruses for the upcoming flu season. The specific strains can vary from year to year. The trivalent vaccine includes antigens from: Influenza A (H1N1), Influenza A (H3N2), Influenza B. However as mentioned earlier WHO is now recommending removal of the influenza B (Yamagata lineage) from such formulations. These vaccines aim to provide protection against the selected strains and help prevent influenza infection. It's worth noting that there are also quadrivalent influenza vaccines that include an additional influenza B strain. WHO determines the vaccine strains to be included in each flu season's vaccine twice a year (once prior to the Northern Hemisphere's flu season and once prior to the Southern Hemisphere's flu season).

### Conjugated recombinant vaccination protocols

Virus entities such as glycoproteins and nucleoproteins or the M2e ion proteins independently are not efficient enough to generate broad-scale immunogenicity and only generate mild passive-type immunological responses by lowering the viral progenies alone in hosts [57]. Therefore, the need is to conjugate these available protocols with adjuvants (discussed below) or with other viral entities to generate a strong immune protective response for controlling influenza epidemics and pandemics [57], [100], [113]. Several approaches are under consideration to induce a multi-carrier or multi-adjuvant conjugation with virus-targeted peptides. In these trials, the lower immunogenic molecules such as M2e proteins are conjugated with virus carrier molecules that may be derived from other viruses such as hepatitis B virus or human papillomavirus proteins, bacterial membrane structure, or artificially manufactured liposomes [100], [114]. Some vaccine candidates such as recombinant M2e carrier vaccines have demonstrated excellent immune protective effects against different influenza strains such as H1N1, H3N2, and H5N1 [114]. However, these conjugated protocols based on M2e conjugates still show lower immunity as compared to NA or HA- based vaccination mediated immune protection.

### VLP vaccines

Conjugation of M2e protein in enveloped VLPs that normally express either HA or NA or both and hence demonstrate excellent immunogenic profiles [115]. These VLP-based vaccines have exhibited better immune responses than whole inactivated virus particles since they lack the shielding impact of large proteins [116], [117]. VLP-based vaccines have shown efficacy against diverse subtypes of influenza including H1N1, H3N2, and H5N1, but suffer from silent amino acid variations in different host species [118]. To overcome this challenge and to improve the vaccine efficacy tandem repeats of M2e epitope sequences for different species have been incorporated into vaccine, which have exhibited better immunogenic profiles and have exhibited cross-protection against virus subtypes even with limited or no adjuvants [115], [118]. However, these viral candidates require further molecular insights and proper vaccination designs involving conjugation with other vaccines, antiviral, and adjuvants to develop an effective broad-spectrum influenza vaccine.

### T cell-based vaccines

Continuous mutation at antigenic sites including HA, NA, and other surface molecules generate influenza variants, which escape the neutralizing antibody response [119]. However, cytotoxic T lymphocytes (CTLs) can recognize the conserved viral structures such NP and PA or the M2e and matrix 1 protein (M1), generating immunity against different influenza subtypes [120], [121]. This mechanism is used for designing different vaccines such as live attenuated, plasmid or DNA vaccines, virus-like articles, and viral vector-based vaccines. These vaccines not only mediate T cell-mediated cytotoxicity against influenza, but also mediate B cell and humoral immune responses against influenza.

### DNA vaccine

In this vaccination protocol, naked plasmids are injected into the host to produce the specific antigens and subsequent antibody-mediated responses. Depending upon the dosages and conjugated adjuvant applications the DNA vaccines could be regulated for their immunogenicity potential [122]. The route of administration plays a critical role in such vaccination protocols. More specifically cutaneous delivery has been largely practiced for its linked benefits of immune cells being abundant in the epidermis, which sufficiently transports the DNA-based vaccine antigens to particular immune cells in lymph nodes [123], [124].

### Prime-boost strategies

Like conjugated and recombinant vaccine protocols, prime-boost strategies are used to infer broad-scale immunity. Such as the co-administration of novel vaccine candidates including virus-like particles, and recombinant protein antigens through an adenovector platform altogether with the conventional vaccine candidate [125]. This priming strategy boosts the serological presence and antibody-based cross-reactivity and immune boosting response [126]. The prime-boost strategy in influenza vaccination involves initially priming the immune system with one formulation of the vaccine, followed by a subsequent booster dose containing a different formulation [125]. For example in Priming Inoculation the initial vaccine includes antigens from specific influenza strains which is followed by the Boosting Inoculation through The booster dose, administered weeks later, contains antigens from influenza strains of different virus strains. The specific timing between the priming and boosting doses may vary, but it's typically designed to allow the immune system to develop a memory response after the priming dose, and the booster serves to enhance and prolong that immune response [126]. The reason behind introducing different strains in the priming and boosting doses, is that the immune system is exposed to a broader range of influenza antigens. This helps create a more diverse and robust immune response [127]. Additionally, the priming dose stimulates the production of memory B and T cells specific to the initial strains. The booster dose then activates these memory cells, leading to a faster and more effective immune response upon subsequent exposure to the virus. Moreover, the prime-boost strategy aims to improve overall vaccine efficacy by addressing the challenge of influenza virus variability [128]. Since Influenza viruses undergo frequent genetic changes, and a prime-boost approach helps enhance protection against a wider spectrum of evolving strains. The booster dose also reinforces and extends the duration of immunity, providing a more prolonged defense against influenza viruses. This strategy is particularly important in the context of influenza, where the virus can undergo frequent antigenic changes, necessitating periodic updates to the vaccine formulations. The prime-boost strategy helps maximize the breadth and durability of the immune response, contributing to more effective protection against diverse influenza strains [129].

The experiments with the primary HA-based DNA vaccines conjugated with the application of season trivalent inactive vaccines have been shown to significantly boost antibody-mediated immune titers [127], [128]. Moreover, these prime-boost strategies are recommended for pandemic incidences to curb the rapid surge of virus infections in the populace [127]. This implication was practiced during the 2009 pandemic in which phase 1 clinical trials were conducted using A(H1N1) [127], [128], [129]. Similarly, DNA prime-MIV boost vaccines against H5N1 and H7N9 also demonstrated improved antibody responses compared to the same vaccines when used alone [88], [129], [130].

### Vaccine adjuvants

Adjuvants act as immunostimulatory agents that help to enhance immunogenicity by promoting innate immunity and inducing humoral or adaptive immune responses when administered along with drugs or vaccine formulations [131]. In addition to immunoregulatory effects, they also add to dose management, desirable responses, and increased memory immune responses in a population that undergoes poor responses upon vaccine such as immunocompromised individuals like the elderly, pregnant women, and children [132]. Moreover, they show immunogenicity against both season and pandemic species of influenza virus and their pharmaceutical, cytotoxic and tolerance profiles are well established for application against influenza and other related virus infections [132], [133], [134]. However, there is still a need to understand the relevant receptors and downstream cellular signaling responses and biodistribution profiles in conjugation with specific antiviral and vaccine formulations [32], [131].

## Discussion: lessons learned from pandemics

During the pandemics of influenza virus, several difficulties and challenges were faced in finding the right antiviral treatment as the approved therapeutics were designed only for acute, common, and uncomplicated cases of influenza infection to reduce symptoms [15], [16], [18], [20]. Rapid resistance development to antiviral drugs further complicated the problem and caused the longevity of the pandemic era [43]. The scientific community was greatly concerned about the resistance and under such conditions, drugs, such as oseltamivir, zanamivir, and peramivir are allowed for seriously ill patients and dosages are limited for acute patients. Oseltamivir and zanamivir were FDA approved in 1999. Peramivir was approved in 2014 with emergency use authorization in 2009 [46], [50], [135]. Moreover, some drug administration routes were also sorted out for severely ill patients such as: oral, intranasal, intravenous, intramuscular. subcutaneous, inhalation, topical and rectal routes. it was also sorted that the specific route of administration for influenza treatment depends on the type of medication, the patient's condition, and the desired effect of the treatment.

New mechanisms have been proposed in terms of therapeutics against host-directed viral replication, such as indirect viral inhibitor. They interfere with the host intracellular process required for viral replication and modulate host immune responses [136], [137]. However, no specific approved drugs have come forward with this mechanism of action, thus more work is needed in this direction. Resistance against antiviral therapies, high mutation rates among influenza viruses, and inability to completely deal with disease symptoms by certain drugs, were the major reasons behind the concept of combination therapy [20], [83], [136], [137]. The basic principle is to aim multiple targets including viral replication machinery, host cell machinery needed for viral replication, adjusting pharmacokinetic potentials of different drug regimens, dealing with the rapid virus resistance patterns and managing immune regulation, especially in complicated immunocompromised case [43], [1]. These combination therapies have been employed for other bacterial and viral diseases such as human immunodeficiency virus (HIV)- where combination therapy under the name of highly active antiretroviral therapy (HAART) has been in trials for a long [46], [82].

The main benefits that are linked with the combination approach are the increase in survival rates, generation of the synergistic and additive effects of combination drugs and reduction in the dosages required for a single drug [65], [43]. A decrease in a single drug dosage also decreases the dose-dependent toxicity and side effects associated with drug usage and reduces the mutational and resistance development among viral strains as well. Different drug combinations are thus being proposed and checked in clinical trials such as double, and triple drug combinations [43], [52], [54], [55], [56], [58], [61]. However, the human’s trials for these therapies are limited owing to the uncertainty associated with viral mutation and drug resistance [[24], [12]. Therefore, it is pertinent to design clinical research trials to specifically evaluate the efficacy of combination therapies in a bigger range of animal models.

Most applicable, licensed, and approved vaccines worldwide are based on traditional methods devised more than 50 years ago, which are facing challenges in terms of viral heterogenicity and the inability to target all virus types by a single vaccine candidate [32]. WHO and CDC regulates semi-annual recommendations to induce strain-specific vaccination trials and clinical practices for different regions of the world [81], [138]. Thus, vaccines are manufactured annually with the updates in line with the exhibited viral mutations in influenza subtypes. The annual practices based on classic vaccine candidates make it difficult to initiate a novel vaccine practice because of the acquaintance with the familiar, cost-effective, consistent, and largely accepted practices of vaccination on old therapeutics [139], [140]. Moreover, the ongoing vaccine trials also face challenges in terms of comparative analyses and placebo models based on traditional therapeutics. However, there is a need to regulate the modern vaccination design if we want to overcome yearly vaccination updates for the coming year ahead. The focus of future vaccination trials should be kept on reducing the morbidity with chronic influenza and mortality rates. For this purpose, regular clinical trials should be carried out to validate the potential universal vaccine candidates.

## Conclusion

The past two decades, especially in the time after the pandemics of 2009–2010, have presented several challenges associated with influenza therapy. For the cause, certain therapies came to the surface as discussed in this review article. These antiviral therapeutics are effective candidates, but all have associated limited efficacy and some side effects and other complications. Moreover, the problem of viral mutations and drug resistance is common to all the discussed therapeutic interventions. Under this scenario, it is essential to practice more on combination therapeutic approaches. Additionally, trials should be conducted to bring out-of-the-box solutions and create competitive therapeutic options that may be based on data from previous therapeutic trials coupled with the latest development in biotechnology.

On the other hand, the recent advances in vaccinology, pharmaceutics, and biotechnology are still stuck in experimental phases. The major issues besides the continuously mutating nature of the virus may involve the technicalities, clinical approvals, ethical considerations, logistic gaps, financial lacking, and regulatory deviations that delay the overall approval procedures. The need of the time is to improvise more qualitative and quantitative research designs and clinical practices to fasten the vaccine approval on an international level. For that, more clinical experiments should be conducted to establish antiviral therapeutics and vaccine efficacious profiles in humans.

**Funding**

M.A. work is supported by Ragnar Söderberg Foundation (M13/18) and Swedish Research Council (2018-02266).

## CRediT authorship contribution statement

**Shiza Malik:** Conceptualization, Data curation, Formal analysis, Investigation, Methodology, Software, Validation, Visualization, Writing – original draft, Writing – review & editing. **Muhammad Asghar:** Conceptualization, Data curation, Formal analysis, Funding acquisition, Investigation, Methodology, Project administration, Resources, Supervision, Validation, Visualization, Writing – review & editing. **Yasir Waheed:** Conceptualization, Data curation, Formal analysis, Funding acquisition, Investigation, Methodology, Project administration, Resources, Supervision, Validation, Visualization, Writing – review & editing.

## Declaration of competing interest

The authors declare that they have no known competing financial interests or personal relationships that could have appeared to influence the work reported in this paper.

## Data Availability

No data was used for the research described in the article.
